# Height Growth Modeling in Ethiopian Children and Adolescents Aged 7–20 Years: A Prospective Cohort Study

**DOI:** 10.1155/bmri/7288345

**Published:** 2025-03-25

**Authors:** Dereje Danbe Debeko, Ayele Taye Goshu

**Affiliations:** ^1^Department of Statistics, Hawassa University, Hawassa, Ethiopia; ^2^Department of Statistics, Cotebe Teaching University, Addis Ababa, Ethiopia

**Keywords:** Ethiopia, growth modeling, height growth, nonlinear mixed effects models

## Abstract

**Background:** Modeling physical growth plays a vital role in examining and defining growth trajectories related to public health and well-being.

**Aim:** The primary objective of this study was to model height growth in Ethiopian children and adolescents aged 7–20 years to estimate the growth variations across the Ethiopian regions.

**Methods:** A total of 891 children and adolescents aged 7.5–20 years were included in the study. To estimate growth spurts within and between study subjects over time, the SITAR and PB1 models were fitted to the height growth measurements gathered in four survey rounds to.

**Results:** Boys experienced puberty 2.6 years later than girls did, while the mean peak height velocity was estimated to be 5.5 cm/year in boys and 6.3 cm/year in girls. The mean adult height in boys was estimated to be 174.6 cm, while in girls, it was estimated to be 162.2 cm. Both girls (*p* < 0.005) and boys (*p* < 0.008) in Amhara and Tigrai regions were significantly shorter compared to their counterparts in Addis Ababa. However, there was no significant height difference between girls and boys in former SNNPRS region, Oromia region, and Addis Ababa. Height at peak velocity strongly correlated with the rate of change during the pubertal period. The rate of change in both boys and girls during the prepubertal and pubertal growth stages was inversely correlated with the adult height.

**Conclusions:** Children who had rapid rate of change during the prepubertal and pubertal periods attained adulthood later in life. There was a significantly different height growth in children and adolescents across the regions of Ethiopia.

## 1. Introduction

Child growth is a key indicator of a child's health and development and has an important influence on future health and well-being [1, 2]. Physical growth in length, for instance, is generally characterized by rapid growth in early life, followed by a general deceleration in the childhood period and a marked increase in the late childhood period along with the onset of puberty [3]. According to Hauspie, Cameron, and Molinari [4], human physical growth can be categorized into four growth phases: 0–2 years with a high rate of growth (just after birth that rapidly decreases); childhood (5–9 years), in which growth is slightly decreasing with a pubertal spurt, which is apparent in some subjects; and puberty (10–18 years), which is the period when growth increases until the subjects achieve maximum growth.

Rapid growth causes individual differences in pubertal development in all growth phases. These differences can be characterized by timing (the relative maturity of a child in relation to their peers of the same sex and age), intensity (the rate at which an individual progresses toward full sexual maturity), and size (the mean height of an individual in relation to the average height of peers) [5, 6]. Human physical growth can, of course, be influenced by genetic factors, parental health, environmental factors, socioeconomic factors, family characteristics, and nutritional status ([7–10]). However, changes in observations and differences that occur between individuals over time are complex and not easy to understand [11].

Researchers have proposed different modeling approaches for both structural and nonstructural growth spurts to understand the changes over time and to capture the complex physical growth changes that occur during the growth process [12]. Most of the proposed modelling approaches, either in structural or nonstructural forms, are beautifully designed to capture human physical growth and development. Conceptually, the basic growth modeling framework is used to capture the average trend or pattern of change over time. Practically, these modeling frameworks fit within individual changes and bridge multilevel modeling traditions with individual differences observed over time [13–18]. However, some of these models are incapable of capturing the real features of growth spurts on the ground [19–23].

Nonlinear models in mixed and/or fixed effects form are widely applied modeling approaches. These modeling techniques characterize the growth spurts and individual-level variation around the population-averaged curves based on heuristic assumptions [24–28]. However, their fitting performance and ability to account for within and between individual growth differences have been tested using growth measurements in developed country settings. Thus, the aim of this study was to model height growth in children and adolescents aged 7–20 years and to estimate height growth variation across the regions of Ethiopia.

## 2. Materials and Methods

This study used longitudinal height growth data from the Young Lives Ethiopian cohort study. The cohort included 3000 children living at 20 sites across Addis Ababa (the capital) and four other regions (Amhara, Oromia, Tigrai, and former Southern Nations, Nationalities, and Peoples' State (SNNPS)) in the country. These cohort studies followed children in two age groups: a younger cohort comprising a total of 2000 children aged 0.5–1.5 years and an older cohort comprising 1000 children aged 7.5–8.4 years at baseline (first round) in 2002. The remaining three rounds of surveys were carried out in 2006, 2009, and 2013 for both cohorts, recpetively. The details of the cohort study can be found on the official website of the project (http://www.younglivesethiopia.org) [29].

We used repeated height growth measurements of older cohort children followed from 2002 to 2013 in four survey rounds. Among the 1000 children and adolescents in the older cohort, 891 subjects whose height was ≥ −2 standard deviations, and those who had no chronic health conditions during each survey round were included in the study. The height in centimeters (measured in four rounds) was used as the response variable. The measurement was done to the nearest 0.1 cm, and age was measured in years with one decimal place. The growth measurements taken in four survey rounds for each subject included in the study as displayed in Figure S5.

### 2.1. Methods of Data Analysis

There have been many growth modeling approaches used to estimate the mean growth rate (fixed effects) or to clearly capture an individual-specific rate of change over time (random effects). However, only some of these models are capable of fitting human physical growth due to their computational tractability, flexibility in capturing the future of physical growth spurts, and interpretability of the parameters.

The Preece–Baines 1 (PB1) and Superimposition by Translation and Rotation (SITAR) models are among the widely applied methods in growth modeling. These models are preferred due to their computational tractability and ability to capture growth spurts with physically meaningful parameters. The SITAR model estimates an individual-specific mean growth curve as a regression B-spline plus a set of up to three fixed and random coefficients. This model is used to simplify a vast array of individual-specific variations into a single median growth curve by taking the adjusted curves and fitting a natural cubic spline to estimate the mean growth curves [30, 31]. The three SITAR model parameters are size (individual mean height compared to average height), timing (individual age at peak height velocity (PHV) (APHV)), and intensity (growth rate compared to average growth). Size, timing, and intensity are estimated for each individual as random effects, and then, the values are adjusted to individual curves with the average value. The model fits data using different numbers of knots (curvatures).

Another structural or parametric form of the growth curve modeling approach is using the PB1 model [32]. This modeling approach has two alternative model forms. The multiplicative exponential-logistic form of the model, which is important to capture growth spurts during adolescent period. Other importance of the PB1 models is its capability of handling timing and tempo and adult size (mean adult height).

### 2.2. Model Fitting: PB1 Model

 
$$\begin{array}{c} y_{\text{ij}}=h_{1}-\frac{2\left(h_{1}-h_{c}\right)}{\exp\left(s_{0}\left(t-c\right)\right.+\exp\left(s_{1}\left(t-c\right)\right.\;} \end{array}$$

where *h*_1_ is the estimated mature or final (adult) height in centimeters, *h*_*c*_ is the height at midpuberty at age *c* (in centimeters), and *c* is the APHV (in year): Peak velocity (PV) is a measure of the maximum rate of growth in height during a growth spurt; APHV corresponds to the maximum growth velocity; *t* is age in years; *s*_0_ and *s*_1_ are prepubertal and pubertal growth rate constants (centimeters/year), respectively.

We used the maximum likelihood method to estimate the model parameters. The fitting performance of the model was tested based on the BIC statistics. Descriptive (table, percentages, and figures) and inferential analyses (parameter estimations) were done using R software (Version 4.3.2) under the “nlme” and ‘sitar' packages. The statistical significance was set at *α* = 0.05.

## 3. Results

### 3.1. Descriptive Statistics

A total of 891 ubjects were included in the study, of which 53% were boys and 47% were girls. Among the subjects included in the study, 13.9%, 20.6%, 20.8%, 23.6%, and 21% were from Addis Ababa, Amhara, Oromia, former SNNPRS, and Tigrai region, respectively (Table 1).

### 3.2. Individual-Specific and Mean Growth Curves

**Figure 1:**
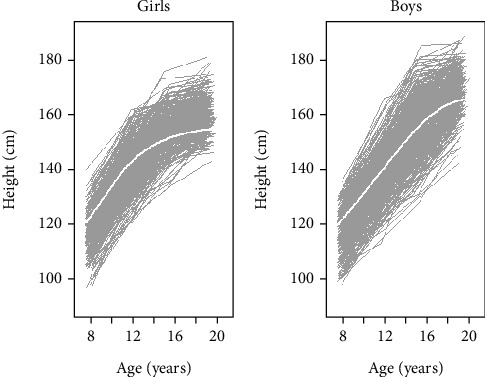
Individual-specific (gray solid lines) and mean height growth curves (white solid line) for (a) 419 girls and (b) 472 boys included in the study (Young Lives Ethiopia, old cohort, 2002–2013).

Individual-specific height growth in boys and girls followed different growth patterns over time. As shown in Figure 1, some girls (Figure 1) and boys (Figure 1) were consistently taller, and others were consistently shorter than the mean height. Some individuals were relatively short at the beginning and become taller, while others do so in the opposite way. In addition, all the girls and boys had a pubertal growth spurt and a time when they grew appreciably faster than before or after.

The growth curves presented in Figure 1 not capable to estimate the rate of change before and during puberty, mean adult height, APHV, height at PV, and the ypes and degrees of relationships between size, timing, and intensity. Thus, we used the SITAR and PB1 models to estimate magnitude and rate of changes in growth spurts within and between the subjects. The fitting performance of the models was done based on the fit statistics, as shown in the next section (Table 2).

### 3.3. Model Compression

The main aim of the model comparisons was to identify the best model fit to the data. The goodness of fit was checked based on the goodness-of-fit statistics (BIC). The test statistics results presented in Table 2 shows that the SITAR model shown best fit to the data used in the study.

### 3.4. SITAR Model

Table 3 contains the standard deviations and correlations of the three SITAR model parameters. Small differences was found between the observed and fitted values (standard residual = 2.82 for girls and 3.62 for boys, respectively). However, individual-level variation in terms of size, timing, and intensity was more dispersed in boys.

**Figure 2:**
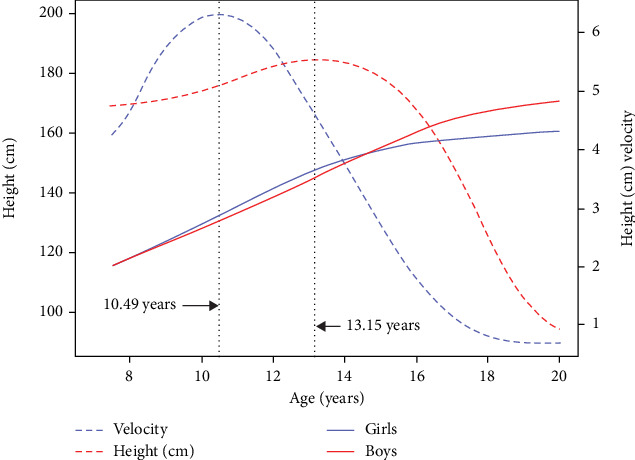
Fitted distance (blue solid line for girls and red solid line for boys) and mean peak velocity curves (blue broken line for girls and red broken line for boys) based on the SITAR model coefficients with respect to age (years).

The area under the velocity curve shown in Figure 2 corresponds to the total increase in size, timing, and intensity. The mean height of the girls surpassed the mean height of the boys, ranging from 9.20 to 14.90 years, and then boys subsequently became taller than the girls. The velocity curve in boys was lower and wider than that in girls, where the mean APHV was estimated to be 10.49 years n girls, while in boys, it was estimated to be 13.15 years. On average, girls reached the APHV 2.66 years earlier than boys did. The growth velocity in girls was estimated to be 6.34 cm/year, while in boys it was estimated to be 5.57 cm/year.

The PV in girls was greater than that of in boys and it was estimated to be 6.34 cm/year in girls and 5.57 cm/year in boys.

### 3.5. Height Growth Velocity Curves Across Regions

**Figure 3:**
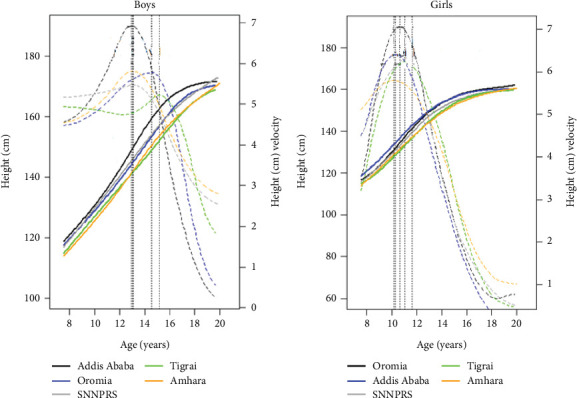
Mean peak height velocity (broken lines) and fitted growth curves (solid lines) of (a) 472 boys and (b) 419 girls by region (Young Lives Ethiopia, old cohort, 2002–2013).

**Figure 4:**
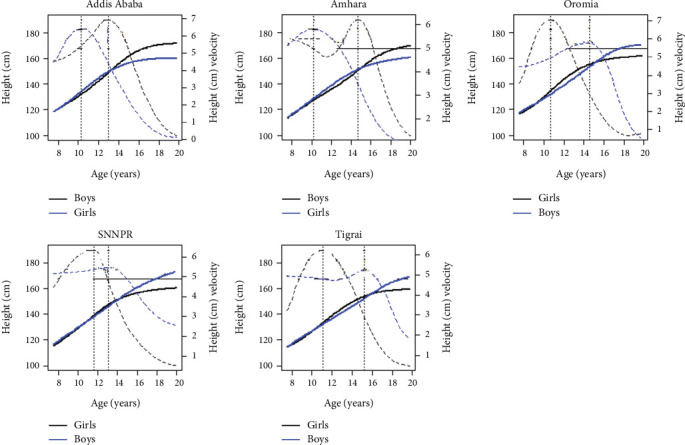
Height growth curves: fitted line (blue solid lines for boys and black solid lines for girls); peak height velocity (blue broken lines for boys and black broken lines for girls) in Oromia, SNNPRS and Tigrai; fitted line (blue solid lines for girls and black solid lines for boys); and peak height velocity (blue broken lines for girls and black broken lines for boys) in the Addis Ababa and Amhara regions.

As shown in the previous section, the estimated PHV was greater in girls than in boys. However, in which region this irregularity occurred was not identified. In this section, we fitted growth curves along with PV for boys and girls across regions using the SITAR model.  Figure 3 illustrates the mean fitted distance and the PV for girls and boys in each region. The fitting distance and PVs in girls and boys by their respective regions are displayed in Figure 4.

Girls in the Oromia region had steeper velocities than girls in other regions. Girls in the Amhara region (PV = 5.8 cm/year) had the least steep velocity, followed by girls in Tigrai region (PV = 6.2 cm/year); in both regions, girls reached PV during similar periods (10.2 and 11.1 years, respectively). In boys, the steepest growth velocity was observed in Addis Ababa (PV = 6.9 cm/year), followed by the Amhara region (PV = 6.2 cm/year). Boys in SNNPRS had the least steep velocity (PV = 5.5 cm/year) compared to their counterparts in other regions. Boys in the Tigrai region experienced puberty later in life (APHV = 15.2 years) and reached PVs 2.3 (Addis Ababa (15.2)–Tigrai (12.9)) and 2.1 (SNNPRS (15.2)–Tigrai (13.1)) years later than boys in Addis Ababa and SNNPRS, respectively. Boys in Addis Ababa experienced puberty earlier in life compared to boys in other regions. This may be due to the better lifestyle and/or access to basic needs compared to other regions of the country. Figure 4 shows the fitted distance and PHV for boys and girls in their corresponding regions. Boys had higher PHV than girls in Addis Ababa and Amhara region. However, PHV in girls was greater than that in boys in Oromia (PV = 7.1 cm/year), SNNPRS (PV = 6.4 cm/year), and Tigrai regions (PV = 6.2 cm/year). This unusual phenomenon might be due to the quality of the data, the small number of data points used for the measurements in girls, the age of the individual subject at the baseline (i.e., younger ages at the time of data collection for boys), and or due to other factors.

The PHV presented in Figures 3 and 4 reveals that there was differences in height growth spurts in children and adolescents across the regions of Ethiopia.

### 3.6. The PB1 Model

Using the SITAR model, we estimated the maximum growth velocity and the corresponding age in boys and girls, as presented in Figures 2, 3, and 4. We used the PB1 model to estimate maximum/adult height, mean age at midpuberty, and prepubertal and pubertal growth rates (Table 4).

The mean adult height in boys was estimated to be 174.6 ± 0.37 cm, while in girls, it was estimated to be 162.2 ± 0.44 cm. Height at PV was 162.24 ± 0.40 cm in boys and 138.70 ± 3.60 cm in girls. APHV in boys was 16.02 ± 1.574 years, while in girls, it was 11.54 ± 0.62 years. The rates of change during the prepubertal and pubertal periods in boys were 0.096 and 0.78 cm/year, respectively, while in girls, it was estimated to be 0.036 and 0.468 cm/year, respectively. In boys, there was a positive correlation between the mean adult height and the height at PV, while in girls, the negative correlation was estimated. However, there was a strong correlation between the rate of change during prepuberty and the pubertal period in both sexes. The height at PV had almost perfect correlation with the rate of change during the pubertal period. For both sexes, the estimated correlation between the mean adult height and the rate of change during the prepubertal and pubertal stages was negative (Table 4).

## 4. Discussion

Modeling, summarizing, and describing the growth process require an optimal approach to understand and observe growth spurts over time. Recently, there has been growing interest in modeling the early life of biological growth in life-course epidemiology. Due to the mathematical tractability and having physically meaningful parameters, the SITAR and PB1 models were apllied to the longitudinally collect height growth measurements in Ethiopian children and adolescents aged 7–20 years. These models were used to estimate size, timing, intensity, prepubertal and pubertal rate of change, maximum/PHV, and corresponding age and to measure the types and degree of the relationship between adult height and rate of change between the prepubertal and pubertal periods.

Based on the SITAR model analysis, girls reached PV 2.6 years earlier than boys did, and APHV was 10.49 and 13.15 years earlier in girls and boys, respectively. A consistent study on height growth in children and adolescents aged 7–19 years in the Northern Finland Birth Cohort (NFBC) [33] revealed that most of the peak height fluctuations typically occurred between the ages of 10 and 14 years and between 12 and 16 years in 95% of girls and boys, respectively. Another study by Pan and Goldstein [34] on the height growth of 156 subjects who were followed from 0.25 to 18.5 years in the Edinburgh Longitudinal Study reported that the PVs in boys and girls were located at 13.8 and 11.5 years of age, respectively. Other findings by Cole and Mori on the mean height growth of children who were followed from 1 to 20 years in South Korea (1965–2005) and Japan (1950–2010) [35] reported that the PV was located at 13.5 years of age in boys and 11.5 years of age in girls. On the other hand, a study by Zemel and Johnston [36], using the longitudinal records of 339 subjects taken from the third Harvard Growth Study, reported that the ages at PHV was approximately 14.1 and 12.2 years for boys and girls, respectively. The current study estimated that mean heights at the PV in boys and girls was 162 cm and 138.7 cm, respectively. Another study by Grimm, Ram, and Hamagami [3] using the Berkeley Growth and Guidance Study reported that the mean height in the middle of puberty was 160.4 cm, which was located at 13.23 years.

The rate of change tended to be constant and became flat after 17 years in girls, whereas the change appeared to increase after 19 years in boys. This finding indicates that the rate of change in girls became constant and reached a maximum height at approximately 17 years of age, while boys reached adult height 19 years later. Adult height was negatively correlated with the rate of change during the prepubertal and pubertal periods in both boys and girls. Adult height was almost perfectly correlated with height during puberty period, negatively correlated with the rate of change before and during puberty period, and negatively correlated with duration puberty period.

A model comparison was performed to test the fitting performances of the models and to identify the best model fit to the data. Based on the fit statistics, the SITAR model, which includes random size, timing, and intensity (velocity), better captured the growth spurts in both sexes. Other studies also compared the fitting performance of growth curve models. According to previous studies, the SITAR model is more flexible, as it does not prefer a particular growth shape to find the shape of the underlying growth curves. Another study by Simpkin et al. [37] concluded that the SITAR and PB1 models are useful methods for adolescent growth modeling because they are capable of providing unbiased estimates of APHV. However, the features of the decision could be dependent on the anthropometric measurement (shape, rate of growth, and clearly identified fixed and/or random parameters incorporated in the models and the age ranges).

## 5. Conclusion

Children who had accelerated rate of change during prepubertal and pubertal periods experienced puberty in early time but reach adult height later in life. There is a significant difference in height growth among children and adolescents across regions of Ethiopia. The PB1 and SITAR mixed effects models could be good choices for modeling height growth spurts in children and adolescents due to their computational flexibility and ability to explore unknown growth curvatures with biologically meaningful parameters.

The current study used height growth measurements of children and adolescents aged 7–20 years. However, these data are not representative of all the regions in the country, and measurements were collected in only four survey rounds. Thus, there might be some effects on the precision of parameter estimation, especially in the case of height velocity in girls, which was unusually greater than that of boys. Therefore, we recommend further cohort studies using cohort data collected from the entire region to validate and better understand the growth spurts of children and adolescents in Ethiopia.

## Figures and Tables

**Table 1:** The number of subjects in the study by region and sex (Young Lives Ethiopia, older cohort, 2002–2013).

**Sex**	**Region**										**Total**	
	**Addis Ababa**		**Amhara**		**Oromia**		**SNNPRS**		**Tigrai**			
	**n**	**%**	**n**	**%**	**n**	**%**	**n**	**%**	**n**	**%**	**n**	**%**
Boys	60	48.4	98	53.3	99	53.2	118	56.2	97	51.8	472	53
Girls	64	51.6	86	46.7	87	46.7	92	43.8	90	48.2	419	47
Total	124	13.9	184	20.6	186	20.8	210	23.6	187	21	891	100

**Table 2:** Goodness-of-fit statistics.

**Sex**	**Models and fit statistics**	
	**Information criteria (BIC)**	
	**SITAR**	**PB1**
Boys	10389	10794
Girls	12071	12090

**Table 3:** The estimated values of the SITAR model random effects coefficients (size, timing, and intensity) and the mean APHV and PHV by sex.

**Model parameters**	**Sex**	**Random effects (Std. dev)**
Size (cm)	Boys	5.15
	Girls	5.50
Timing (years)	Boys	1.28
	Girls	1.29
Intensity (%)	Boys	0.12
	Girls	0.17
Residual	Boys	3.63
	Girls	2.82

**Table 4:** Summary of estimated values of the PB1 model coefficients by sex.

			Boys (*n* = 472)		Girls (*n* = 419)			
Fixed effects		Fixed effects			
Parameters			Estimates	Std. error	Estimates			Std. error

Adult height (*h*_1_ in cm)			174.60	0.38	162.2			0.44
Height at peak velocity (*h*_*c*_ in cm)			162.24	0.40	138.70			3.60
Prepubertal growth rate (*s*_0_ in cm/year)			0.096	0.0003	0.04			0.002
Pubertal growth rate (*s*_1_ in cm/year)			0.78	0.004	0.47			0.004
Age at peak velocity (*c* in years)			16.02	1.57	11.54			0.62

Correlation					Correlation			
	*h*_1_	*h*_*c*_	*s*_0_	*s*_1_	*h*_1_	*h*_*c*_	*s*_0_	*s*_1_

*h*_*c*_	0.36				−0.64			
*s*_0_	−0.57	0.53			−0.64	0.99		
*s*_1_	−0.67	0.28	0.86		−0.81	0.94	0.92	
*c*	0.30	0.68	0.16	0.004	−0.63	0.99	0.98	0.94

## Data Availability

The data that support the findings of this study are openly available at Oxford University at https://www.younglives-ethiopia.org/.

## Supporting Information

Supporting Information Additional supporting information can be found online in the Supporting Information section. Figure S5: Height growth measurements taken during each survey rounds for each individual included in the study (419 girls and 472 boys).
