# Supplementary material for: Height Growth Modeling in Ethiopian Children and Adolescents Aged 7–20 Years: A Prospective Cohort Study
Source: Biomed Res Int. 2025 Mar 25;2025:7288345. doi: 10.1155/bmri/7288345 (PMC11961283; doi:10.1155/bmri/7288345)
Supplement: Supporting Information — Additional supporting information can be found online in the Supporting Information section. Figure S5: Height growth measurements taken during each survey rounds for each individual included in the study (419 girls and 472 boys). [file 7288345.f1.docx]

Supplementary material


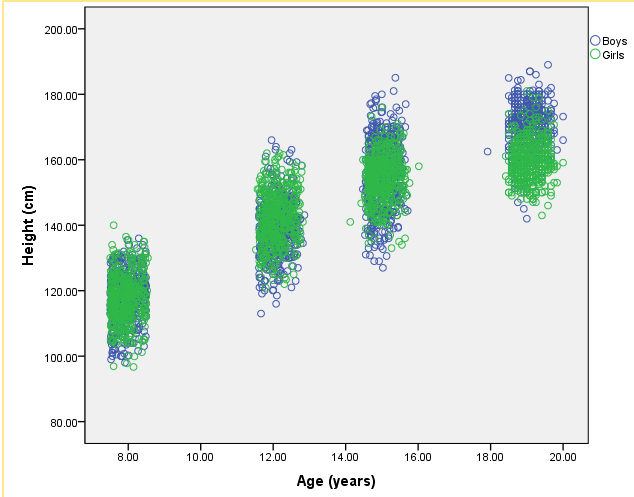


Supplementary Figure: Raw data of height growth measurements taken in four rounds for 419 girls and 472 boys taken from Young Lives Ethiopia, older cohort followed from 2002 to 2013
